# Superparamagnetic Iron Oxide Nanoparticles as a Tracer for Sentinel Lymph Node Mapping in Endometrial Cancer

**DOI:** 10.3390/ijms26020781

**Published:** 2025-01-17

**Authors:** Marcin A. Jedryka, Piotr Klimczak, Marcin Kryszpin, Tymoteusz Poprawski, Andrzej Czekanski, Piotr Lepka, Rafał Matkowski

**Affiliations:** 1 Department of Oncology, Wroclaw Medical University, 50-367 Wroclaw, Poland; andrzej.czekanski@gmail.com (A.C.); piotrlepka@yahoo.pl (P.L.); rafal.matkowski@umw.edu.pl (R.M.); 2 Department of Oncologic Gynecology, Lower Silesian Oncology, Pulmonology and Hematology Center, 53-413 Wroclaw, Poland; krysza2@wp.pl (M.K.); tymoteuszpoprawski@gmail.com (T.P.); 3 Department of Gynecology and Obstetrics, Provincial Specialist Hospital, 64-100 Leszno, Poland; petros69@op.pl; 4 Breast Cancer Clinic, Lower Silesian Oncology, Pulmonology and Hematology Center, 53-413 Wroclaw, Poland

**Keywords:** sentinel lymph node, lymphadenectomy, endometrial cancer, superparamagnetic iron oxide, nanoparticles, tracer

## Abstract

Sentinel lymph node (SLN) detection has been widely investigated in recent years as a part of the surgical staging of women with endometrial cancer (EC), gradually overtaking lymphadenectomy (LND) in this respect. In this study, thirty EC patients, assumed as stage I, were investigated using superparamagnetic iron oxide (SPIO) as a tracer for SLN detection followed by LND. The endpoints of this research were the proportion of successful SLN detection, the average number of SLNs per patient, the percentage of bilaterally detected SLNs, and the proportion of metastatic SLNs. Safety endpoints were the summary of all reported adverse events. SLNs were detected in all cases and bilaterally in 21 patients (70%). The diagnostic accuracy parameters of the SPIO detection of metastatic SLNs evaluated by Receiver Operating Characteristic (ROC) curve analysis with the area under the ROC curve (AUC) demonstrated a sensitivity of 80% and AUC of 0.9 (*p* < 0.001), confirming the SPIO technique’s efficacy in women with EC. No adverse events were reported. SPIO nanoparticles as a tracer for SLN mapping in apparent early-stage EC patients demonstrated satisfactory accuracy parameters and safety; however, these data need to be evaluated by further research.

## 1. Introduction

Endometrial cancer (EC) is the most common gynecological tumor in developed countries, with its incidence continuing to rise [1]. For 2024, it was estimated that more than 7782 women would suffer from EC in Poland and more than 2553 patients would die from this disease [2].

EC spreads mostly via the lymphatic system; hence, lymph node dissection (LND) has become an integral part of the standard surgical approach for this cancer [3]. However, such an extensive surgical procedure may lead to significant drawbacks such as lymphedema or vascular and nerve injury. Sentinel lymph nodes (SLNs) are the nodes that first receive lymphatic drainage from the tumor area, and these lymph nodes (LNs)are most likely to harbor metastasizing cancer cells when lymphatic metastases occur [4]. Over the past ten years, SLN mapping for EC has been widely studied regarding the use of different tracers and injections sites [5,6,7]. Further studies have established contemporary algorithms for SLN mapping generally based on the clinical use of an indocyanine green (ICG) tracer with near-infrared imaging systems [5,8,9].

An alternative SLN detection system is based on nanoparticles of superparamagnetic iron oxide (SPIO) that respond to an external magnetic signal produced by a miniaturized detector [10,11]. The SPIO agent, covered with biocompatibile molecule, has been demonstrated clinically as a safe and efficient contrast for magnetic resonance imaging (MRI) when administered intravenously [12,13]. The carboxydextran coating prevents SPIO agglomeration while maintaining biocompatibility. The Z-averaged nanoparticle diameter, including the organic coating is 60 nm. This diameter enables SLNs to selectively filter out the particles [14]. After injection into the interstitial tissue, SPIO nanoparticles naturally drain to LNs via the lymphatic system where they are physically filtered, trapped, and concentrated due to macrophage phagocytosis [10,11,15]. SPIO exhibits superparamagnetic behavior, characterized by a response to an external magnetic field while retaining no magnetic remnant in its absence. This specific feature makes SPIO nanoparticles ideal for SLN detection, as their collective moment can be used to detect the nodes. Their brownish appearance acts as a visual stain aiding intra-operative identification.

The feasibility of SLN detection using SPIO has been well documented in many prospective trials concerning breast cancer [10,11,15,16,17,18] and other malignancies, such as prostate cancer [19] and vulvar cancer [20,21]. However, the efficacy of the SPIO technique in EC has yet to be investigated. Therefore, we decided to study the accuracy parameters of the SLN superparamagnetic mapping procedure with the use of a portable magnetic field detector operated by a surgeon and followed by LND as the standard of care in apparent early-stage EC patients.

## 2. Results

In total, we assessed the eligibility of 35 patients for this study; however, only 30 cases were included in the analysis. Two patients were excluded because of a violation of the study protocol; the SLN procedure was performed with a two-day delay due to the patients’ poor condition, which postponed our schedule. However, we were still able to read the signals with the magnetometer and find SLNs in these patients, confirming the long-lasting effect of the SPIO injection in the cervix due to prolonged concentration in the lymphatic system. Three patients had intra-operatively found enlarged lymph nodes that had not been described by MRI at the time of preoperative assessment, causing the surgeons to withdraw from the SLN study protocol.

We found SLNs in every case (the overall detection rate was 100%); however, they were detected bilaterally in 21 patients (70%). In total, 90 SLNs were detected and excised. In total, 80 SLNs (89%) were located in pelvic lymph nodes (PLNs), while 10 (11%) were found among para-aortic lymph nodes (PALNs). Of note, 88 SLNs (97%) were described as brownish (due to the SPIO coloration). The proportion of SLNs detected per patient was, on average, 3.0 lymph nodes (range 1–8). During the systematic LND, we removed 605 nodes in total, resulting in an average of 20.2 lymph nodes per patient (range 18–79). The mean signal count was read as 1634 (1634 ± SD), with a mean background count after SLN excision of 26 (11 ± SD). The median (IQR) SPIO detection signal count from SLNs was 1250 (680–2020) vs. a 25 (20–30) count read from LND (Table 1). There were no serious adverse events reported.

Histopathological examination showed six positive SLNs (with micrometastases) in four patients and twelve positive LNs from subsequent LND in five patients (three with macro- and nine with micrometastases), of whom four had positive SLNs alike.

The false-negative case with negative pelvic SLNs but with positive PALNs (non-SLNs) demonstrated an endometrioid low-grade tumor located in the uterine fundus with a deep myoinvasion and substantial presence of lymphovascular space involvement (LVSI). The distribution, localization, and accompanying clinicopathological features of the patients with metastatic LNs are presented in Table 2.

The general malignancy rate for patients was 16.6%, while for nodes it was 2.9%. The malignancy detection rate (MDR) for SLNs per patient was 80% and 33% per node. A discordant detection of metastatic lymph nodes between the SLN biopsy and LND was shown in one patient with negative pelvic SLNs but positive PALNs; the false-negative rate (FNR) was calculated as 16%. However, in 29 of the 30 study cases, the data were concordant; thus, the concordance rate was 96.6%. The statistical analysis showed a sensitivity of 80% regarding metastatic SLN detection compared to LND. Both the specificity and positive predictive value (PPV) were 100%, while the scores for the negative predictive value (NPV) and accuracy were 97.8% and 98%, respectively (Table 3).

**Figure 1:**
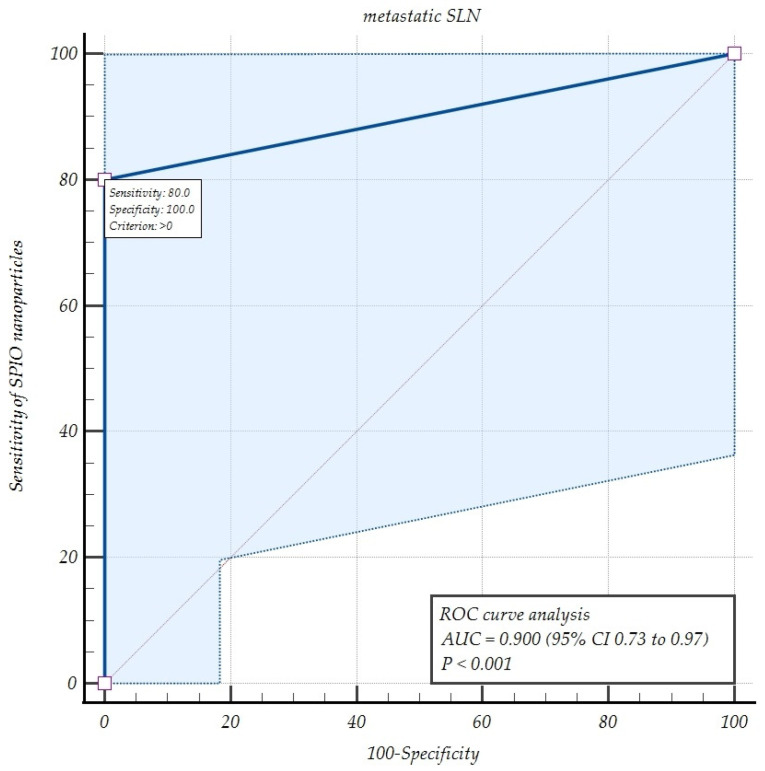
The accuracy parameters of the superparamagnetic iron oxide detection of metastatic sentinel lymph nodes evaluated by Receiver Operating Characteristic (ROC) curve analysis. Abbreviations: SPIO—superparamagnetic iron oxide; SLN—sentinel lymph node; AUC—Area Under Curve.

The diagnostic accuracy parameters of the SPIO detection of metastatic SLNs evaluated by Receiver Operating Characteristic (ROC) curve analysis with the area under the ROC curve (AUC) demonstrated a sensitivity of 80% and AUC of 0.9 (*p* <0.001), confirming the SPIO technique’s efficacy in women with EC (Figure 1).

## 3. Discussion

In this study, we assessed the efficacy of SPIO nanoparticles injected intra-cervically and traced with a magnetic field detector for SLN identification in EC patients, followed by LND as a recommended standard of care and the study control. We found SLNs in every case of the study cohort; however, the bilateral detection rate was demonstrated as 70%. The general malignancy rate was 16.6%; the MDR for SLNs was 80% at the patient level and 33.3% at the nodal level. We demonstrated a sensitivity of 80% confirmed by the ROC curve analysis, NPV of 90%, and FNR of 16% for this new SLN mapping technique.

Sensitivity is the crucial parameter for determining the efficacy of the SLN concept and can only be assessed reliably when the procedure is followed by a systemic LND that can detect any metastasis in the lymphatic basin of the malignancy-afflicted organ. In order to estimate the accuracy of SPIO as a novel tracer for SLN detection in EC patients, we compared our research results to other studies of commonly used tracers that reported their findings in comparison with a LND regarding detection rate (both overall and bilateral), sensitivity, NPV, and FNR (Table 4).

The first prospective trial concerning the SLN technique in EC patients presented 100% sensitivity and NPV per hemipelvis based on the cervical injection of a blue dye and radioisotope (technetium 99 (Tc^99^)) followed by LND [22]. However, the detection rate per patient decreased to 84% and NPV to 97%, displaying three missed LN metastases in the presence of negative SLNs [27]. Another prospective, multi-institutional study included EC patients that had undergone an SLN biopsy followed by complete LND with a standardized technique (a robotic surgical platform with ICG as a tracer) [7]. The authors presented a 97.2% sensitivity, an NPV of 99.6%, and an FNR of 2.8%. However, the bilateral detection rate was rather low (52% vs. 70% in our study with SPIO), which was explained as a learning curve effect of a new surgical technique. The feasibility and accuracy of the SLN technique in EC patients was confirmed by a large meta-analysis study [4]. The SLN detection rate ranged from 23% to 100%, with a pooled average of 81% (compared to 100% in our study); the pooled-average bilateral detection rate was 50% (range of 6% to 88%) vs. 70% in this research. The mean number of SLNs detected per patient was 2.9compared to 3 SLNs in our study, with a para-aortic pooled-average SLN detection rate of 17%, almost twice as high as our result (9%). Tumor histology (endometrioid vs. non-endometrioid), grading, average patient body mass index (BMI), surgical approach, and study size were not significantly associated with the SLN detection rate [4]. In our study, the overall SLN detection rate was 100%; therefore, study population clinicopathological parameters such as age, BMI, endometrial tumor histological type, tumor size and grading, and LVSI should not affect the result. The study patients with metastatic SLNs similarly presented various clinical and pathological features (Table 2), suggesting that they did not affect the malignancy detection rate. However, those correlations were not calculated statistically due to the limited number of cases with metastatic SLNs. Finally, the discussed meta-analysis showed that the pooled sensitivity of the SLN detection of metastases was 96% (95% CI, 93–98), with a pooled NPV of 99.7% [4]. In comparison, our results were inferior, with a sensitivity of 80% and NPV of 97.8%. We consider that a para-aortic SPIO distribution could be impaired in the presence of a metastatic disease in the region and thus influence the SLN detection rate. One case from our study cohort presented negative pelvic SLNs and LNs but positive para-aortic LNs (without identifying any SLNs in the para-aortic region). Additionally, a deep myometrial infiltrating tumor was located in the uterine fundus; thus, an ovarian-like lymphatic spread could be predominant in that patient. In the literature, the SLN detection technique in EC was criticized for not fully evaluating PALNs for metastases, especially after a cervical tracer injection [27,28]. However, such an isolated para-aortic nodal metastasis was found only in 2–3% of patients, and that result was further reduced to 1.5% with an ultrastaging processing of apparently negative pelvic SLNs [29]. In our study, we applied a pathological ultrastaging protocol for SLNs, although it did not reveal any metastases in that particular case. Sometimes, SLN detection may be impaired by an LN metastasis that disturbs lymph flow. The SPIO/MRI technique, combined with iron staining, in cases of metastatic LNs, demonstrated a decrease in iron uptake and contrast defects [30]. Thus, due to the PALN macrometastases in our study, the nodal iron load could be insufficient to reveal those involved LNs as positive SLNs, resulting in the abated sensitivity of our data. Nevertheless, the reason for false-negative para-aortic SLNs is that the recommended surgical algorithm of EC nodal staging accepts a small percentage (1–3%) of missed isolated para-aortic LN metastases, leaving to the surgeon the decision of whether to perform a para-aortic LND following negative para-aortic SLN mapping after thoroughly applying all the steps of the staging scheme [31].

To our knowledge, this is the first study to assess the feasibility of the SPIO technique for SLN mapping in early-stage EC patients. Our results in terms of the overall and bilateral detection rate, sensitivity, and NPV support the use of this superparamagnetic technique, not only in superficially localized malignancies with a well-defined lymphatic spread but also in the tumors of organs with a more complex anatomy, such as endometrial cancer.

The weaknesses of our study comprise its single-institution, low-volume study cohort and a clinical heterogeneity that could bias the overall accuracy results. However, the study design of our preliminary research, including the small population, resulted from the initial assumption that superparamagnetic nanoparticles may be of use for SLN detection in early-stage EC patients without further distinctions. Considering our SLN false-negative case with positive PALNs, we suggest that the SPIO technique for open abdomen surgery could be preceded by preoperative MRI mapping (after the SPIO injection) to minimize the risk of omitting metastatic LNs. Ultimately, though, we consider the SPIO detection system potentially helpful in the SLN biopsy procedure for apparent early-stage EC patients in the case of open surgery (so far, no endoscopic magnetometer has been manufactured) being necessary instead of minimally invasive techniques for any medical reasons, particularly in institutions that already possess this device and have experience with its use in the SLN biopsy of other malignancies. Minimal invasive surgery is a dedicated procedure for SLN mapping in early-stage EC; however, massive adhesions after numerous surgeries or pelvic inflammatory diseases and/or accompanying serious comorbidities can make such a technique not feasible and safe for a patient. The cervical route of the SPIO nanoparticles’ administration is convenient and comparable to other tracer injection techniques; however, it was considered uncomfortable or even painful by our patients. SPIO tracer collection in SLNs produces simple-to-identify visible and audible signals, aided with SLN colorization. Such a technique could at least be equal in its ease of application compared to the radiotracer and/or dye SLN mapping procedures used during open surgery, especially due to its simplicity and efficacy without logistical issues such as access to nuclear medicine facilities or costly equipment with near-infrared detection systems. Furthermore, the safety of the patients and staff is guaranteed as this technique avoids exposure to ionizing radiation, and last but not least, in our country, SLN detection is significantly less expensive with a superparamagnetic iron oxide tracer than the standard radioisotope or ICG dye. However, further prospective and comparative studies with a larger study population are required.

## 4. Materials and Methods

This preliminary and observational study was performed in the oncological gynecology department of the Lower Silesian Oncology, Pulmonology and Hematology Center in Wroclaw, collaborating with the Wroclaw Medical University, Poland. The study was approved by the Wroclaw Medical University Bioethics Committee (registration no: KB-356/2019, dated 9 May 2019) and conducted in accordance with the Declaration of Helsinki. Patients were enrolled between September 2019 and May 2023.

### 4.1. Study Protocol

The study inclusion criteria were as follows: a diagnosis of primary apparent early-stage EC with a surgical intervention treatment plan, namely total abdominal, extrafascial hysterectomy with bilateral salpingo-oophorectomy (TAH/BSO) and an SLN biopsy procedure followed by pelvic and para-aortic LND, regarded as the standard of care, and the study control; a clinically and radiologically (MRI) negative LN status (N0); no evidence of distant metastases (M0); and the written informed consent of an adult subject to participate in the study. The exclusion criteria were as follows: a suspicion of metastases, including enlarged and suspected LNs; preoperative radiotherapy to the affected uterus; an iron overload disease; intolerance or hypersensitivity to iron oxide; a metal implant close to the expected SLN location; a surgical intervention treatment plan involving minimally invasive surgery; a lack of written consent; mental deterioration; and pregnancy and lactation in menstruating women.

### 4.2. Patients

The final study cohort included 30 EC patients (2 peri- and 28 postmenopausal) with an average age of 65.5 years (range 47–80) and body mass index (BMI) of 30.5 kg/m^2^ (range 21.4–37.5). The postoperative pathologic report showed an average tumor size of 51 mm in diameter (range 10–95) and an infiltrated myometrium more than 50% of its width in 20 cases. The tumor histology was endometrioid in 25 patients, and high-grade tumors were determined in 10 patients, including 5 cases of non-endometrioid cancer. A substantial LVSI was established in 11 of the 30 studied women.

### 4.3. Procedure Description

**Figure 2:**
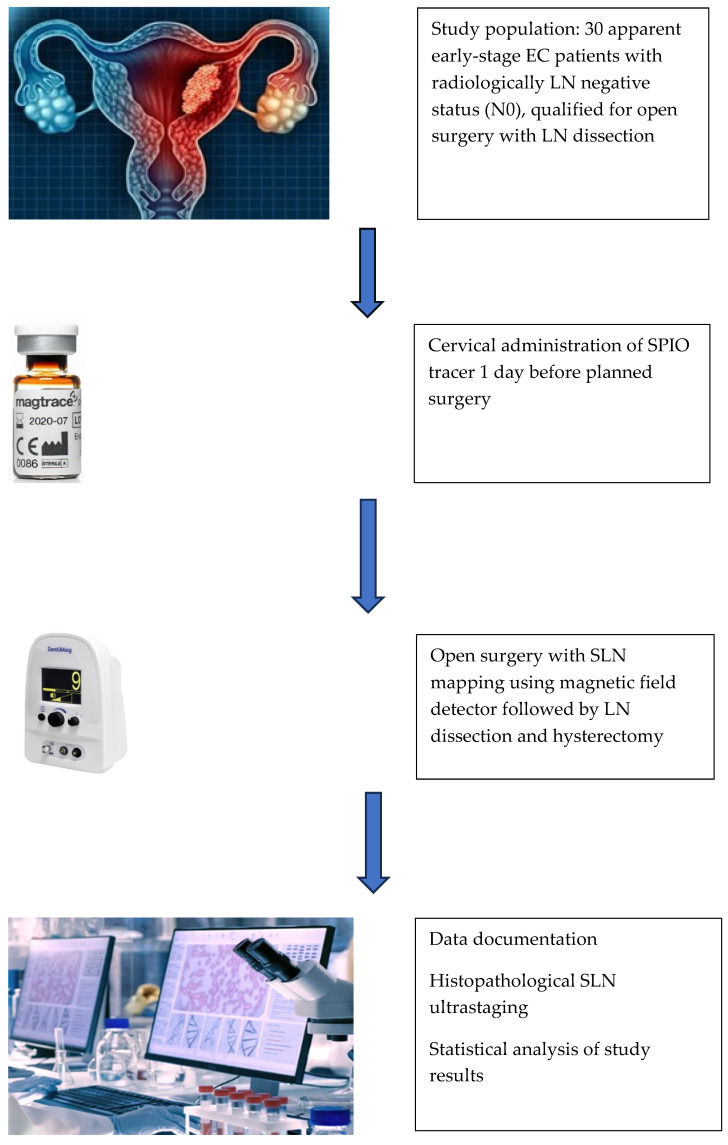
Schematic illustration for the procedure of SPIO nanoparticles administration (Magtrace^®^, Endomagnetics Ltd., Cambridge, UK) and SLN mapping with a magnetic field detector (SentiMag^®^, Endomagnetics Ltd.,Cambridge, UK) in the study patients. Abbreviations: EC—endometrial cancer; SPIO—superparamagnetic iron oxide; LN—lymph node; SLN—sentinel lymph node.

One day before the planned surgery date, a 2 mL vial of undiluted SPIO manufactured for standard use as a tracer (Magtrace^®^, Endomagnetics Ltd., Cambridge, UK) was injected with a 23 G needle about 1–3 mm under the cervical epithelium and 10–15 mm deep into the cervix interstitially at 3 and 9 o’clock (1 mL each side, 0.5 mL for each superficial and deep injection). Then, the standard open surgery technique for an abdominal hysterectomy and lymphadenectomy was performed with an assessment of abdominal and pelvic organs to rule out macroscopic metastases, followed by retroperitoneal access along the large vessels from the obturator fossa on both sides up tothe left renal vein. After the removal of any metal instruments from the proximity of LNs, they were thoroughly scanned with a magnetic field detector/magnetometer (SentiMag^®^, Endomagnetics Ltd., Cambridge, UK) to find tracer signals. The rule was to excise every LN as an SLN if the count reading was at least >10% of the node with the highest reading from the magnetometer. After excision, each marked SLN was measured ex vivo with the SPIO tracer detection device to confirm the in vivo read and documented (signal values, anatomical localization and brown coloration). Then, SLNs were sent for a histopathological assessment including our institutional pathology ultrastaging protocol. When no more SLNs were found, the standard procedure of an LND followed by TAH/BSO was performed. All removed LNs during a systemic LND created our control. The specimens were examined by two experienced pathologists via a routine EC pathological protocol adopted in our institution. The study scheme is illustrated in Figure 2.

### 4.4. Statistical Analysis

The primary endpoint of this study was the proportion of SLNs successfully detected with the SPIO technique followed by LND as the standard of care. The secondary endpoints were average SLNs per patient, the percentage of bilaterally detected SLNs, and the proportion of pathologically positive results (malignancy detection rate—MDR) per patient and per node. The general malignancy rate was defined as the percentage of patients with LN metastases from the total studied population. Sensitivity described the proportion of patients with metastatic LNs who had positive SLNs, NPV defined the proportion of patients with negative SLNs who had no metastases, and FNR expressed the proportion of patients with metastatic LNs who presented negative SLNs or failed to identify the disease with the study tracer. The safety endpoints were the summary of all reported adverse and serious events, especially related to the SPIO detection procedure.

The study data were statistically calculated and described as the mean ±SD and median (interquartile range (IQR)). We used STATISTICA version 13.3 (TIBCO Software Inc., Palo Alto, Ca, USA). Detection rates were estimated at both the patient and node level. The SPIO tracer nodal detection rate indicated the sensitivity and PPV, while the data from the LND presented the true negatives. Hence, specificity and NPV could be assessed. The diagnostic performance of the SPIO detection test to discriminate metastatic SLNs from metastatic LNs was evaluated using ROC curve analysis with AUC, depicting how the SPIO technique was distinguished between these study cohorts.

## 5. Conclusions

This preliminary study of the SPIO nanoparticle technique for SLN mapping in apparent early-stage EC patients demonstrated satisfactory accuracy parameters of detection rate, sensitivity, NPV, and patients’ safety related to this novel tracer. The efficacy of metastatic SLN detection with the use of the SPIO method in comparison with the standard of care (LND) was confirmed by the ROC curve analysis. These results are encouraging considering SLN mapping in the population of women with endometrial carcinoma qualified for open abdomen surgery instead of minimally invasive procedures; however, they need to be evaluated by further research.

## Figures and Tables

**Table 1:** Superparamagnetic iron oxide detection counts in dissected lymph nodes from women with endometrial cancer. Abbreviations: SLN_ALL—all sentinel lymph nodes; SLN_PLN—all pelvic sentinel lymph nodes; SLN_PALN—para-aortic sentinel lymph nodes; LND—lymphadenectomy.

Statistical Analysis	SLN_ALL Count	SLN_PLN Count	SLN_PALN Count	LND Count
Mean	1634	1593	2082	26
Median	1250	1655	1379	11
SD	1634	1250	1925	25
25% centile	680	660	860	20
75% centile	2020	1746	3240	30
Interquartile range	1340	1086	2380	10

**Table 2:** Clinicopathological characteristics of the study cases with metastatic lymph nodes. Abbreviations: SLN—sentinel lymph node; LND—lymphadenectomy; LVSI—lymphovascular space involvement; MIC—micrometastasis; MAC—macrometastasis; LG—low grade; HG—high grade.

Clinicopathological Features	Case 1	Case 2	Case 3	Case 4	Case 5
Metastatic SLN (*n*)	1	3	1	1	0
Metastasis type	MIC	MIC	MIC	MIC	
Localization	internal iliac right	1 and 2—common iliac, left; 3—external iliac, left	internal iliac, right	internal iliac, left	
Metastatic LND (*n*)	3	3	1	2	2
Metastasis type	1—MIC; 2—MAC; 3—MIC	MIC	MIC	MIC	MAC
Localization	1—external iliac, right; 2—paracaval; 3—aortocaval	1 and 2—common iliac, left; 3—paracaval	internal iliac, right	1—internal iliac, left; 2—para-aortic	1—paracaval; 2—aortocaval
Tumor type	carcinosarcoma	endometrioid	carcinosarcoma	endometrioid	endometrioid
Tumor size (mm)	30	53	50	60	45
Grading	HG	HG	HG	LG	LG
Myometrial invasion (superficial) (≤50%)			present		
Myometrial invasion (deep) (>50%)	present	present		present	present
LVSI	positive	positive	positive	negative	positive

**Table 3:** Sentinel lymph node detection efficacy (with the use of superparamagnetic iron oxide as a tracer) compared to standard lymphadenectomy in endometrial cancer patients. Abbreviations: SLN—sentinel lymph node; LND—lymphadenectomy.

Statistical Analysis	Value (%)	95% CI
Sensitivity	80.0	28.3% to 99.4%
Specificity	100.0	86.7% to 100.0%
Positive Predictive Value	100.0	39.7% to 100.0%
Negative Predictive Value	97.8	88.6% to 99.6%
Accuracy	98.0	85.2% to 99.9%
Malignancy Rates	per patient (%)	per node (%)
Malignancy Rate (general)	16.6	2.9
Malignancy Detection Rate for SLN	80.0	33.3

**Table 4:** Comparison between studies on the accuracy of sentinel lymph node detection with different tracers in endometrial cancer patients and the results of the present study using superparamagnetic iron oxide nanoparticles (SPIO study). Abbreviations: SPIO—superparamagnetic iron oxide; ICG—indocyanine green; Tc99—radioisotope technetium 99.

Study	Patients (*n*)	Tracer	Sensitivity (%)	Bilateral Detection Rate (%)	Negative Predictive Value (%)	False Negative Rate (%)
Ballester et al. [22]	125	Blue Dye + Tc99	84	69	97	3
Rossi et al. [7]	293	ICG	98	52	98	5
Papadia et al. [23]	42	ICG	90	88	97	10
Touhami et al. [24]	128	ICG, Tc99, Blue Dye, Blue Dye + Tc99	96	74	98	4
Soliman et al. [25]	101	ICG, Tc99, Blue Dye, Blue Dye + Tc99	95	58	98	5
How et al. [26]	100	Blue Dye + Tc99	89	62	99	8
SPIO Study	30	SPIO	80	70	98	16

## Data Availability

Data will be made available on request.
